# Withanolides from *Withania aristata* as Antikinetoplastid Agents through Induction of Programmed Cell Death

**DOI:** 10.3390/pathogens8040172

**Published:** 2019-10-01

**Authors:** Atteneri López-Arencibia, Desirée San Nicolás-Hernández, Carlos J. Bethencourt-Estrella, Ines Sifaoui, María Reyes-Batlle, Rubén L. Rodríguez-Expósito, Aitor Rizo-Liendo, Jacob Lorenzo-Morales, Isabel L. Bazzocchi, José E. Piñero, Ignacio A. Jiménez

**Affiliations:** 1 Instituto Universitario de Enfermedades Tropicales y Salud Pública de Canarias, Universidad de La Laguna, Avda. Astrofısico Fco. Sanchez, S/N, 38203 La Laguna, Tenerife, Canary Islands, Spain; atlopez@ull.edu.es (A.L.-A.); unidesi@hotmail.com (D.S.N.-H.); carlosbethencourtestrella@gmail.com (C.J.B.-E.); ines.sifaoui@hotmail.com (I.S.); mreyesba@ull.edu.es (M.R.-B.); ruben_rguez_exposito92@hotmail.com (R.L.R.-E.); aitorrizo@gmail.com (A.R.-L.); jmlorenz@ull.edu.es (J.L.-M.); 2 Departamento de Obstetricia y Ginecología, Pediatría, Medicina Preventiva y Salud Pública, Toxicología, Medicina Legal y Forense y Parasitología, Universidad de La Laguna, 38200 La Laguna, Tenerife, Spain; 3 Instituto Universitario de Bio-Orgánica Antonio González, Departamento de Química Orgánica, Universidad de La Laguna, Avenida Astrofísico Francisco Sánchez 2, 38206 La Laguna, Tenerife, Canary Islands, Spain; ilopez@ull.edu.es

**Keywords:** *Leishmania*, *Trypanosoma*, *Withania aristata*, withanolides, apoptosis-like

## Abstract

Leishmaniasis and American trypanosomiasis are parasitic diseases that cause significant clinical, social and economic impact on the population of tropical and subtropical countries. Their current treatment is limited and presents multiple drawbacks, including high toxicity, high cost, lengthy treatment plans, as well as the emergence of resistant species. Therefore, there is a need to find new lead compounds with high potency against parasites and low toxicity in patients. In the present work, the bioguided fractionation of an endemic plant from the Canary Islands, *Withania aristata*, led to the identification of withanolide-type metabolites (**1**–**3**) with leishmanicidal and trypanocidal activities. Compounds **1** and **3** showed a significant dose-dependent inhibition effect on the proliferation of *L. amazonensis* promastigotes and *T. cruzi* epimastigotes, higher than the reference drugs, miltefosine and benznidazole, respectively. Moreover, compounds **1**–**3** were more potent (IC_50_ 0.055–0.663 µM) than the reference drug against the intracellular amastigote stage of *L. amazonensis,* with a high selectivity index on murine macrophage cells (SI 58.66–216.73). Studies on the mechanism of death showed that the compounds induced programmed cell death or that which was apoptosis-like. The present findings underline the potential of withanolides as novel therapeutic antikinetoplastid agents.

## 1. Introduction

Leishmaniasis and American trypanosomiasis (Chagas disease), protozoal diseases, are neglected tropical diseases causing considerable morbidity, affecting millions of people every year worldwide [1]. Leishmaniasis is an infectious disease caused by protozoal parasites of the genus *Leishmania* and is transmitted by the bite of a sand fly belonging to the genera *Lutzomyia* and *Phlebotomus*. Leishmaniasis presents three different forms: cutaneous, mucocutaneous and visceral [2]. Furthermore, American trypanosomiasis affects nearly 8 million people in endemic countries and the available treatments only include benznidazole or nifurtimox, which are effective only in the acute phase of the infection [3]. Despite advances in antiparasitic chemotherapy and supportive care, these diseases have remained a public health challenge, mostly due to problems associated with chemotherapeutic regimen. In fact, the treatment of these infections has been ineffective by variable sensitivity between species, toxicity, route of administration, requirement for long courses of administration, resistance, adverse effects and cost [4]. In this sense, natural products from plants are a promising source of novel lead compounds, characterized by unique chemical architectures, pharmacophores and inherent drug-like properties [5].

The genus *Withania* (Solanaceaea) is distributed in the Macaronesian region, the east of Mediterranean area, and south Asia [6], and some species are well known in folk medicine. The therapeutic potential of *Withania* species has been ascribed to the presence of withanolides, which are structurally diverse C28-steroidal compounds with an ergostane skeleton [7]. In particular, *Withania aristata* is an endemic plant from the Canary Islands, popularly known as *orobal*. This plant has been used in traditional medicine as a scarring agent, antispasmodic, as well as for rheumatism, eye diseases and otitis, insomnia, constipation and urinary pathologies [8]. Previous phytochemical studies on *W. aristata* described the isolation of withanolide-type metabolites with cytotoxic [9,10] and diuretic properties [11], including the well-known antiproliferative agent withaferin A [12].

Several typical markers of mammalian apoptosis have been found in *Leishmania*, suggesting the existence of an apoptosis-like death in this genus. These markers include: cell shrinkage, nuclear chromatin condensation, DNA fragmentation, membrane blebbing, mitochondrial transmembrane potential loss and phosphatidylserine exposure [13]. Moreover, *Leishmania* cell death appears very peculiar, since different stimuli can induce a form of cell death with the same phenotypic features as in mammalian apoptosis that could be named *Leishmania* apoptosis [14] or apoptosis-like.

The present work reports the identification of three known withanolides from the leaves of *W. aristata* through a bioassay-guided fractionation carried out against *Leishmania* spp. and *Trypanosoma cruzi*. In order to investigate the mechanism of action for their antiparasitic effects, a group of experiments was performed to study the induction of the apoptosis-like mechanism.

## 2. Results and Discussion

### 2.1. Bioassay-Guided Fractionation

**Scheme 1:**
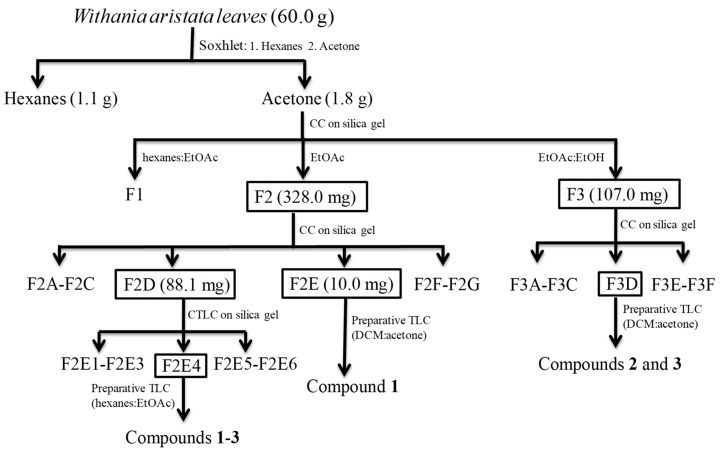
Flowchart of the kinetoplasticidal bioguided fractionation of *Withania aristata* leaves.

The hexanes and acetone extracts of the leaves of *Withania aristata* were evaluated against promastigote forms of *L. amazonensis* and *L. donovani*, and on the epimastigote stage of *T. cruzi*. Cytotoxicity on murine macrophages was also assessed searching for selectivity (Table 1). In addition, miltefosine and benznidazole were evaluated for comparative purposes as reference drugs against leishmaniasis and Chagas disease respectively. Miltefosine shows IC_50_s of 2.64 µg/mL and 1.35 µg/mL against *L. amazonensis* and *L. donovani*, respectively, and CC_50_ of 29.42 µg/mL, whereas benznidazole showed an IC_50_ of 1.81 µg/mL against *T. cruzi*, with a CC_50_ of 104.1 µg/mL. The acetone extract showed activity against the three tested species, with IC_50_s between 2.87 and 20.25 µg/mL. The most sensitive species was *L. amazonensis* and the less sensitive one was *L. donovani*. The selectivity index (SI), ranging from 2.1 to 14.9, was a promising fact to continue with the bioassay-guided fractionation. Therefore, the acetone extract was submitted to vacuum liquid chromatography on silica gel affording three fractions, F1–F3 (Scheme 1). The most active fractions, F2 (IC_50_ values ranging from 1.02 to 12.73 µg/mL) and F3 (IC_50_ values ranging from 3.63 to 22.28 µg/mL), exhibited potent activity on *L. amazonesis* and *T. cruzi*, similar to the reference drugs. Furthermore, both fractions showed a good selectivity index (SI around 11.0) on murine macrophages (Table 1), highlighting these two fractions as the most promising ones.

The most active fractions, F2 and F3, were further chromatographed on a silica gel column yielding seven (F2A–F2G) and six sub-fractions (F3A–F3F), respectively, which were assayed against the three parasite species. From Fraction 2, F2D exhibited a potent antikinetoplastid effect on *L. amazonensis* and *T. cruzi,* which was also higher than, and similar to, miltefosine (IC_50_ 0.36 versus 2.64 µg/mL) and benznidazole (IC_50_ 1.73 versus 1.81 µg/mL), respectively. Regarding fraction F3, sub-fraction F3D was the most active one on the three parasites species, showing IC_50_s of 1.01 and 3.67 µg/mL for *L. amazonensis* and *L. donovani*, respectively, and an IC_50_ of 3.59 µg/mL for *T. cruzi*. In addition, the active fractions showed a moderated cytotoxic profile, with CC_50_ values (>7.65 µg/mL) high enough to continue with the phytochemical analysis.

**Figure 1:**
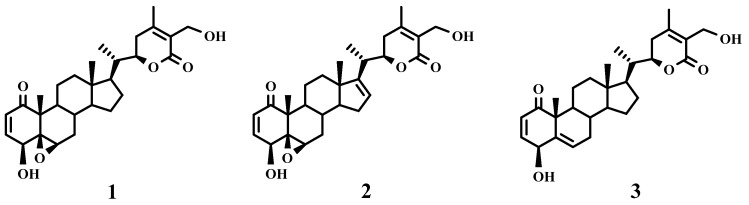
Chemical structure of withanolides **1**–**3** isolated from *Withania aristata*.

Therefore, sub-fraction F2D was submitted to purification steps, affording the known withanolides **1**–**3** (Scheme 1, Figure 1). Moreover, sub-fraction F2E showed to be the most potent one on *L. amazonensis* (IC_50_ value 0.19 µg/mL), which after preparative TLC yielded compound **1**. Furthermore, sub-fraction F3D was submitted to preparative TLC, affording withanolides **2** and **3** (Scheme 1). Their chemical structures were identified as withaferin A (**1**) [10], 4β,17α,27-trihydroxy-1-oxo-witha-2,5,24-trienolide (**2**) [9] and witharistatin (**3**) [10] by spectrometric and spectroscopic data, including 1D and 2D NMR experiments, and comparison with data reported in the literature.

Compounds **1**–**3** were evaluated against the three parasite species (Table 2). The results, over the promastigote stage, showed that compounds **1** and **3** were 7.8- and 2.3-fold more potent than the reference drug [15] against *L. amazonensis* (IC_50_ 0.83 and 2.82 µM, respectively). Moreover, compounds **1** and **3** were 6.8- and 2.9-fold more potent than benznidazole against *T. cruzi* epimastigotes (IC_50_ 1.02 and 2.41 µM, respectively). Regarding the selectivity index (SI) on macrophages, as shown in Table 2, compound **1** has a potent effect on *L. amazonensis* (SI 14.36 versus 11.14 for miltefosine).

Moreover, activity of the withanolides (**1**–**3**) against the intracelular amastigote stage of *L. amazonensis* (Table 3) was higher than that of miltefosine (IC_50_ 3.12 µM), showing IC_50_ values ranging from 0.055 to 0.663 µM. In fact, withanolides **1** and **3** were 56- and 14.9-fold more potent than the reference drug. Besides, the three compounds showed a higher selectivity index than miltefosine (Table 3), showing compound **1** to have an SI of 216.73 versus 23.13 for miltefosine.

### 2.2. Mechanisms of Cell Death

The programmed cell death or apoptosis-like death, is crucial for parasite development and pathogenesis, and the ability of a drug to modulate the life or death of a parasite is recognized for its immense therapeutic potential [16]. In this work, we carried out several experimental approaches to research the apoptotic potential of the withanolides **1**–**3** on *L. amazonesis* and *T. cruzi*.

#### 2.2.1. Withanolides induced Mitochondrial Damage in *L. amazonensis*

**Figure 2:**
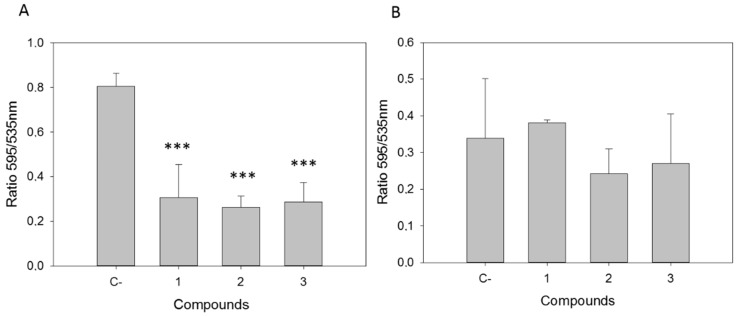
Changes in the mitochondrial membrane potential (ΔΨm) after 24 h of incubation with the IC_90_ of withanolides **1**–**3** in (**A**) *L. amazonensis* and (**B**) *T. cruzi*. C-: Control. Error bars represent the standard deviation (SD). Each data point indicates the mean of the results of three measurements (***) *p* < 0.001.

**Figure 3:**
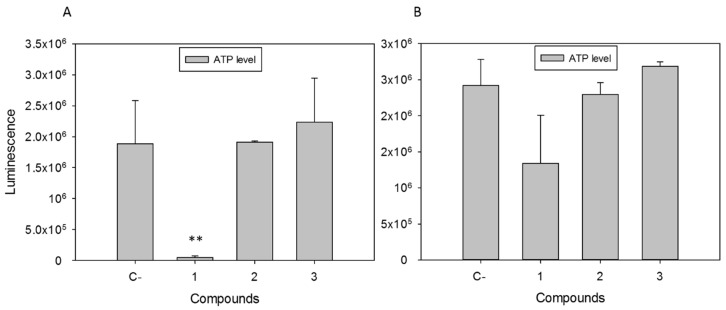
ATP levels in relative luminescence units of (**A**) *L. amazonensis* and (**B**) *T. cruzi* after 24 h of incubation with the IC_90_ of compounds **1**–**3**. C-: Control. Error bars represent the standard deviation (SD). Each data point indicates the mean of the results of three measurements (**) *p* < 0.01.

The effect of the tested compounds on the mitochondrial membrane potential was determined by JC-1 fluorescence measure. Moreover, the tested compounds induced a noticeable decrease in the depolarization of *L. amazonensis* mitochondrial membrane (ΔΨm), since the JC-1 dye remained in the cytoplasm in its monomeric form (Figure 2), thereby showing no clear effect on *T. cruzi*. The mitochondrial damage was confirmed by quantifying the ATP level after 24 h. Compound **1** at IC_90_ produced a strong decrease in the total ATP level for *L. amazonensis* (Figure 3). Furthermore, this effect was not seen in *T. cruzi,* with an ATP level similar to the untreated cells and with a slight decrease when incubated with compound **1**.

Due to *T. cruzi* epimastigotes being seemingly unaffected at the mitochondrial level by the assayed compounds, analysis of the cell death mechanism was continued only in the *L. amazonensis* species.

#### 2.2.2. Withanolides Caused Chromatin Condensation in Treated Cells

**Figure 4:**
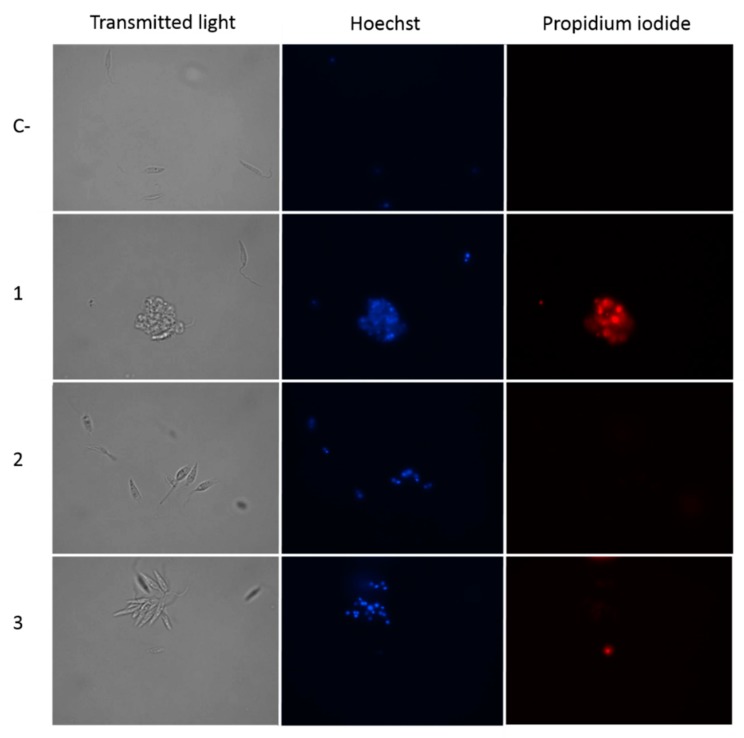
Hoechst-propidium iodide staining: *L. amazonensis* incubated with IC_90_ of withanolides **1**–**3** and the chromatin condensation was analyzed after 24 h. Images (100×) show chromatin condensation (blue) in treated cells. Red fluorescence corresponds to the propidium iodide stain. C-: Control. Images were obtained using an EVOS FL Cell Imaging System.

In view of the obtained results, we decided to analyze the chromatin condensation event, a hallmark of apoptosis-like death, in which small and compact chromatin nuclei appear. To this end, parasites of *L. amazonensis* were incubated for 24 h with the IC_90_ of the withanolides, and subsequently, stained with Hoechst and propidium iodide. Condensation of chromatin in the parasites was clearly observed by fluorescence microscopy, since the treated cells showed a higher amount of bright blue in the nucleus (Figure 4). In addition, propidium iodide staining in red was observed in parasites of *L. amazonensis* treated with compound **1**, indicating an advanced process of death, as confirmed by the transmitted light image, in which parasites appeared to be visibly damaged.

#### 2.2.3. Withanolides Induce Cytoplasmic Membrane Permeability in Treated Cells.

**Figure 5:**
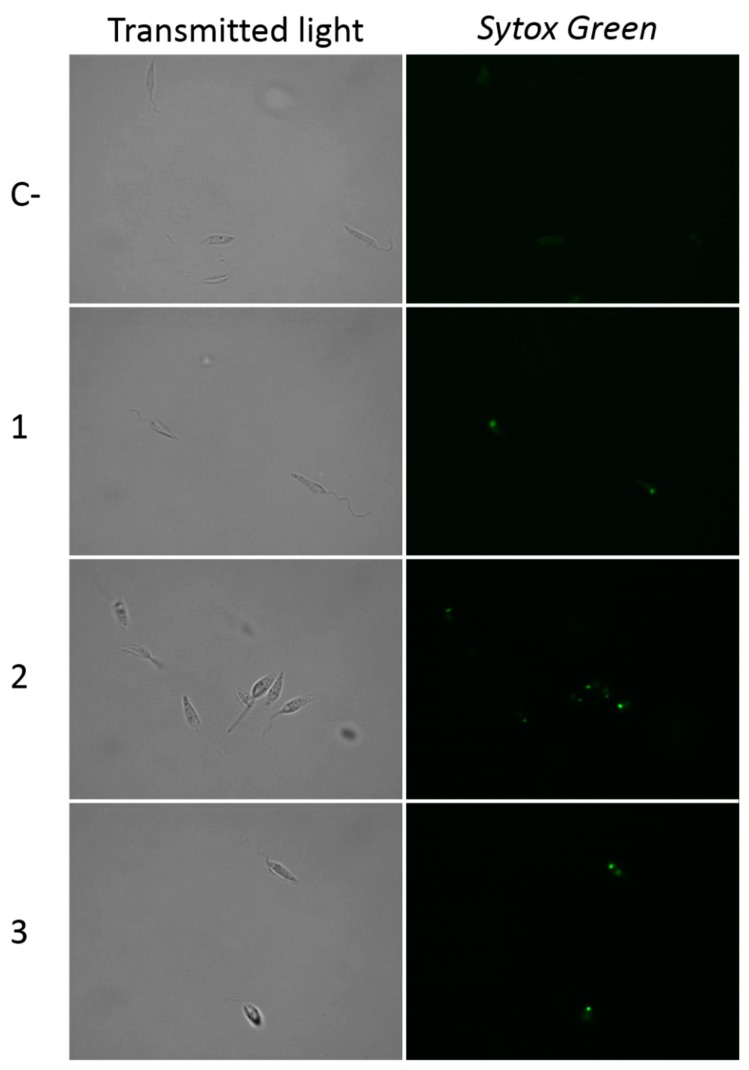
Plasma membrane permeability alteration (Sytox^®^ Green) caused by withanolides **1**–**3**. C-: Control. Images (100×) are representative of the cell population observed in the performed experiments. Images were obtained using an EVOS FL Cell Imaging System.

The cytoplasmic membrane permeability assays on promastigotes of *L. amazonensis* with withanolides **1**–**3** (Figure 5) showed green fluorescence inside the cells, indicating that the plasmatic membrane permeability was slightly damaged, since the cells preserved their integrity and shape.

#### 2.2.4. Withanolides Induce Oxidative Stress in *L. amazonensis*

**Figure 6:**
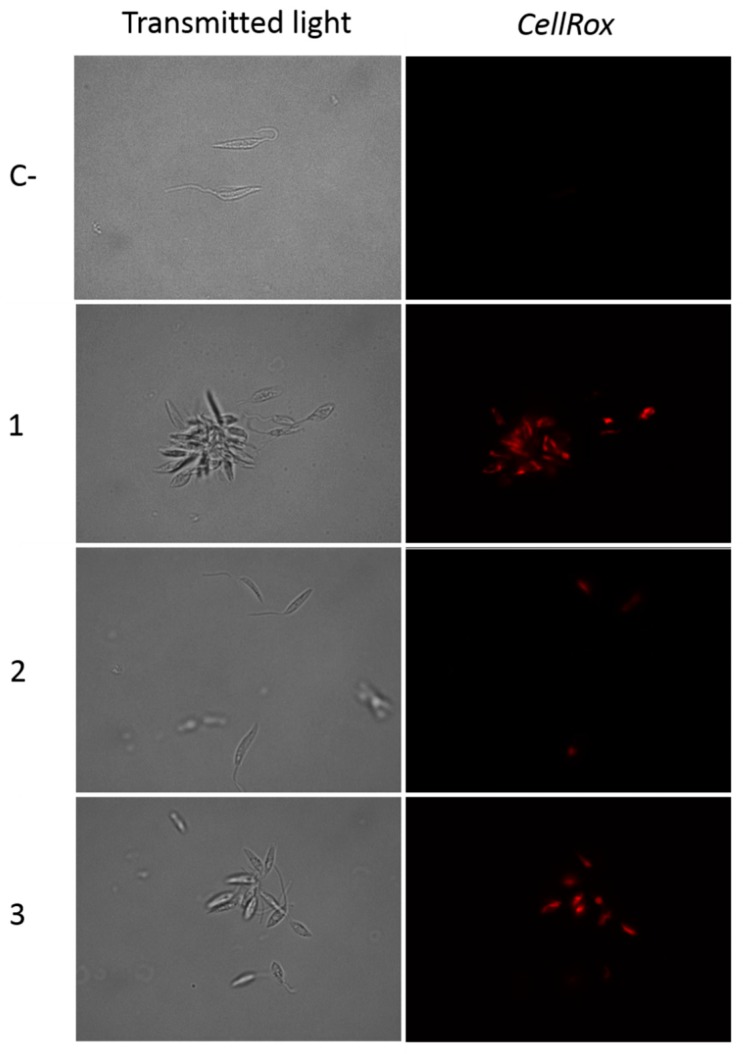
CellROX Deep Red staining. Results after 24 h of incubation of *L. amazonensis* promastigotes with the IC_90_ of withanolides **1**–**3**. C-: Control. Images were captured using an EVOS FL Cell Imaging system (Thermo Fisher Scientific, Waltham, MA, USA) (100×).

As could be observed in Figure 6, after 24 h of incubation of *L. amazonensis* promastigotes with the IC_90_ of the withanolides **1**–**3**, the treated cells showed a higher amount of red staining in the cytoplasm. This corresponds with the reactive oxygen species (ROS) accumulation inside the cell, a well-established event on cells before undergoing a programmed cell death. ROS induced by chemotherapeutic agents is closely associated with mitochondrial function, cytochrome c release, and the apoptosis-like mechanism.

Some species belonging to the *Withania* genera have been shown to present antileishmanial activity. One of the most studied is *Withania somnifera,* a well-known plant in the Ayurvedic medicine system, which extracts exhibited activity against *L. donovani* parasites on in vivo models as well as against *L. major* promastigotes and intracellular amastigotes [17,18]. *Withania coagulans* aerial parts demonstrated activity against *L. major* [19]. Furthermore, to the best of our knowledge, only a study on the evaluation of *Withania somnifera* against *T. cruzi* has been reported [20].

Withanolides have attracted considerable attention due to their potential in drug research and development [7]. In particular, withaferin A, a well-known anticancer drug candidate, exerts its anticancer effect via induction of apoptosis in several human cancer cells [21]. Recent studies have revealed that withaferin A-analogues are potent apoptotic inducers in different tumor cell lines, evidenced by DNA fragmentation, chromatin condensation and phosphatidylserine exposure, indicating an apoptosis mechanism of action in cancer cells [22,23]. Moreover, the leishmanicidal activity of withaferin A has been reported against *L. donovani,* inducing an apoptosis-like cell death mechanism by inhibiting protein-kinase C, leading to the depolarization of mitochondrial membrane potential and releasing of cytochrome c into the cytosol, which induces formation of ROS inside cells, causing oxidative DNA lesions [24]. Our data agree with these previous studies, because depolarization of mitochondrial membrane potential was detected, as well as accumulation of ROS, DNA condensation and a lack of ATP, that proves the mechanism of apoptosis-like in *L. amazonensis* induced by withaferin A (**1**), as well as by compounds **2** and **3**. These compounds could induce apoptosis-like through the mitochondrial pathway initiated by ROS production. Therefore, our proposal is that withanolides-type compounds could induce the formation of ROS, leading this excessive ROS to trigger apoptosis-like death by altering the mitochondrial membrane potential and damaging the respiratory chain, as has been proven in a human leukemia cell line [25].

## 3. Material and Methods

### 3.1. General Procedures

Optical rotations were recorded at 25 °C on a Perkin Elmer 241 automatic polarimeter in CHCl_3_ and the [α]_D_ values are given in 10^−1^ deg cm^2^/g. IR (film) spectra were recorded on a Bruker IFS 55 spectrophotometer. NMR spectra were performed on Bruker Avance 500 and 600 spectrometers at 300 ⁰K. The EIMS and HREIMS data were obtained on a Micromass Autospec spectrometer. HRESIMS (positive mode) were performed on an LCT Premier XE Micromass Electrospray spectrometer. Silica gel 60 (particle size 15–40 and 63–200 μm, Macherey-Nagel) was used for column chromatography (CC), while silica gel 60 F254 was used for analytical thin layer chromatography (TLC). Centrifugal planar chromatography was performed by a Chromatotron instrument (model 7924T, Harrison Research Inc., Palo Alto, CA, USA) on manually coated silica gel 60 GF254 (Merck, Kenilworth, NJ, USA) using 1, 2, or 4 mm plates. The developed TLC plates were visualized by UV light and then spraying with HOAc-H_2_SO_4_-H_2_O (80:16:4), followed by heating at 100 °C for 3 min. All the used solvents were purchased from Panreac. Reagents, deuterated solvents and benznidazole were provided by Sigma-Aldrich. For bioassays, Schneider’s medium (Sigma-Aldrich, St. Louis, MO, USA), Alamar Blue^®^ reagent (Invitrogen, Life Technologies, Carlsbad, CA, USA), EnSpire^®^ Multimode Plate Reader (Perkin Elmer, Waltham, MA, USA), RPMI 1640 and LIT media (Gibco, Thermo Fisher, Madrid, Spain), and Leika DMIL inverted microscope (Leika, Wetzlar, Germany) were used. Miltefosine, used as a reference drug, was purchased from Æterna Zentaris, Charleston, SC, USA.

### 3.2. Plant Material

*W. aristata* leaves were harvested in Icod de los Vinos (Tenerife, Canary Islands, Spain). A voucher specimen (TFC 53219) is stored at the Herbarium of the Department of Botany, University of La Laguna, Tenerife. The identification was performed by MSc Cristina González Montelongo.

### 3.3. Extraction, Bioassay-Guided Fractionation and Isolation

The air-dried powdered *W. aristata* leaves (60.0 g) were extracted successively with hexanes and acetone in a Soxhlet apparatus. The organic extracts were concentrated under reduced pressure to give hexanes (1.1 g, 1.8%) and acetone (1.8 g, 3.0%) extracts. Both extracts were assayed against two species of *Leishmania* spp. promastigote form and epimastigote stage of *T. cruzi* for their anti-protozoal activity. After the preliminary screening, the active acetone extract was subjected to a bioassay-guided fractionation procedure. In this way, the extract was fractioned by liquid chromatography on silica gel, eluted with hexanes/EtOAc (2:8, 0.5 L), EtOAc (0.5 L) and EtOAc/EtOH (8:2, 0.5 L), affording three fractions (F1–F3). The most active F2 fraction (328.0 mg) was chromatographed on silica gel column eluting with mixtures of increasing polarity of hexanes/EtOAc (2:8 to 0:10) and EtOAc/EtOH (10:0 to 9:1) to yield 39 sub-fractions, which were combined on the basis of their TLC profile in seven sub-fractions (F2A to F2G). Sub-fraction F2D (88.1 mg) was chromatographed on silica gel by centrifugal thin-layer chromatography (CTLC) on a 1 mm plate, using mixtures of dichloromethane (DCM)/acetone from 9:1 to 7:3) as eluent to give six sub-fractions (F2D1 to F2D6). Sub-fraction F2D4 (19.4 mg) was further purified by preparative TLC with hexanes/EtOAc (1:10) to yield compounds **1** (11.2 mg), **2** (1.9 mg) and **3** (2.4 mg). Sub-fraction F2E (10.0 mg) was further purified by preparative TLC with DCM/acetone (8.5:1.5) to yield compound **1** (7.3 mg). Fraction F3 (107.0 mg) was chromatographed on a silica gel column eluting with mixtures of increasing polarity of hexanes/EtOAc (9:1 to 0:10) and EtOAc/EtOH (10:0 to 8:2) to yield 31 sub-fractions, which were combined on the basis of their TLC profile in six sub-fractions (F3A–F3F). Sub-fraction F3D (7.2 mg) was further purified by preparative TLC with DCM/acetone (8:2) to yield compounds **2** (4.1 mg) and **3** (2.9 mg). The structures of the compounds were identified as withaferin A (**1**) [10], 4β,17α,27-trihydroxy-1-oxo-witha-2,5,24-trienolide (**2**) [9] and witharistatin (**3**) [10] by NMR spectroscopy and mass spectrometry, and comparison with data reported in the literature.

### 3.4. Cultures

To perform the experiments, promastigotes of *L. amazonensis* (MHOM/BR/77/LTB0016) and *L. donovani* (MHOM/IN/90/GE1F8R) and epimastigote *T. cruzi* (Y strain) were employed. *Leishmania* species were cultured in Schneider’s medium (Sigma-Aldrich, Madrid, Spain) at 26 °C supplemented with 10% fetal bovine serum (VWR, Biowest, Nuaillé, France) as well as in RPMI 1640 medium (Gibco, Thermo Fisher, Madrid, Spain). Epimastigotes of *T. cruzi* were cultured in Liver Infusion Tryptose (LIT) medium supplemented with 10% fetal bovine serum at 26 °C. For the cytotoxicity assays, murine macrophage cell line J774A.1 (ATCC TIB-67) was maintained at 37 °C in a 5% CO_2_ atmosphere in RPMI 1640 medium supplemented with 10% fetal bovine serum.

### 3.5. Leishmanicidal and Trypanocidal Assays Cytotoxic Effect Evaluation

#### 3.5.1. Leishmanicidal Activity Assay

Promastigote in logarithmic phase were used for plate preparation, and the in vitro antiprotozoal assay was performed in 96-well plates. The different fractions or compounds were serially diluted on the wells and 10^6^ parasites/mL were finally added. The activity of the fraction was calculated by the alamarBlue^®^ method [26]. In addition, the active pure molecules were tested against the amastigote stage of *L. amazonensis*.

The amastigote activity was performed according to Jain et al. [27]. After allowing the transformation of rescued amastigotes to promastigotes, the activity was evaluated by AlamarBlue method. After incubation, the fluorescence was measured in a Perkin Elmer EnSpire spectrofluorometer and fluorescence was measured at emission peak 585 nm.

#### 3.5.2. Trypanocidal Capacity Assay

For the evaluation against the epimastigote stage of *T. cruzi*, assays were performed following the same procedure mentioned in Section 3.5.1., modifying the density to 2 × 10^5^ parasite/well and using LIT medium.

#### 3.5.3. Cytotoxicity Assay

The cytotoxicity was evaluated using a macrophage cell line J774A.1 in RPMI medium. Macrophages were plated and at 10^5^ macrophages/well and, subsequently, serial dilution of samples were added to the plates. After 24 h of incubation cell viability was determined by the alamarBlue^®^ [28].

### 3.6. Mechanisms of Cell Death

#### 3.6.1. Mitochondrial Membrane Potential evaluation

To perform the experiment, JC-1 Mitochondrial Membrane, Potential Assay Kit, Cayman Chemical, was employed according the instructions of the kit. Red and green fluorescence was measured with an Enspire microplate reader (PerkinElmer, Waltham, MA, USA).

#### 3.6.2. Measurement of ATP

To determine the ATP level of the parasites, the Cell Titer-Glo^®^ Luminescent Cell Viability Assay (Promega, Madison, WI, USA) was used following the manufacturer’s instructions after incubation of the parasites with the calculated IC_90_ of the tested withanolides for 24 h.

#### 3.6.3. Chromatin Condensation Determination

The detection of condensed chromatin, was performed using the Vybrant™ Apoptosis Assay Kit #5, Hoechst 33342/Propidium Iodide (Invitrogen, Carlsbad, CA, USA). Parasites were incubated with the IC_90_ of the pure compounds for 24 h, then collected and centrifuged. The cell pellet was resuspended in RPMI, added to a black plate and incubated with the Hoechst 33342 and the propidium iodide. Then the plate was protected from light for 20 min at 4 °C. The EVOS FL Cell Imaging System (Invitrogen, ThermoFisher, Carlsbad, CA, USA) was used to observe the cells, using the DAPI (Hoechst) and RFP (PI) Light Cubes.

#### 3.6.4. Plasma Membrane Permeability

In order to detect membrane permeability alterations SYTOX^®^ Green dye was used. Briefly, 10^6^ parasites/mL previously incubated with the IC_90_ of the compounds for 24 h, were incubated with SYTOX^®^ Green (Molecular Probes, Eugene, OR, USA) at 1 μM. The fluorescence was monitored in an EVOS FL Cell Imaging System AMF4300, Life Technologies, USA. This dye is impermeable to intact cells, but when their membrane permeability is changed, SYTOX Green has the capacity to get inside the cell and attach to DNA, increasing its fluorescence >500 times.

#### 3.6.5. Oxidative Stress

CellRox Deep Red Oxidative Stress Reagent (Thermo Fisher Scientific, Waltham, MA, USA) is a probe designed to measure reactive oxygen species (ROS) in live cells, exhibiting strong fluorogenic signal under oxidative state. Following the manufacturer’s protocol, parasites were incubated with the compounds at the IC_90_ concentration for 24 h, then washed, and incubated with 5 μM of CellRox Reagent for 30 min at 26 °C. Then, parasites were centrifuged and resuspended in buffer. H_2_O_2_ at 600 μM for 30 min was used as positive control [29]. A fluorescence microscope (EVOS FL) was used to observe the cells. The signal for Deep Red is localized in the cytoplasm.

### 3.7. Statistical Analysis

Data are presented as mean ± SE. Non-linear regression analysis was used for the IC_50_ (inhibitory concentration 50) and CC_50_ (cytotoxic concentration 50) calculations. All determinations were performed in triplicate and the data shown are representative results from at least three independent experiments. Statistical differences between means were tested using one-way analysis of variance (ANOVA; three or more samples), using the SigmaPlot 12.0 software. A significance level of *p* < 0.05 was used.

## 4. Conclusions

In summary, the bioassay-guided fractionation of *W. aristata* against *Leishmania* spp. and *T. cruzi* was employed to identify drug candidates for the treatment of leishmaniasis and/or Chagas disease. We have successfully identified three potent and selective withanolides with significant leishmanicidal profiles, and high selectivity indexes for *L. amazonensis*. In addition, withanolides induced several ultrastructural and morphological changes in promastigotes of *L. amazonensis*. Mitochondrial membrane potential changes, ROS production, phosphatidylserine externalization, cell shrinkage, a rounded shape of the parasites, nuclear condensation and no change in membrane permeability were observed in parasites that were treated with the mentioned molecules. The major finding in the present study was that these withanolides induced programmed cell death in *L. amazonensis*, sharing several phenotypic characteristics with other cases of programmed cell death in metazoans, and exhibiting them as promising candidates against leishmaniasis. These findings support future investigations for further biochemical studies to unveil the mechanism of action of these withanolides, which can lead to the optimization of treatment for leishmaniasis.

## Figures and Tables

**Table 1:** Activity ^a^ against promastigote stage of *Leishmania* spp., epimastigote stage of *T. cruzi*, and cytotoxicity against eucariotic cells of the extract, fractions and sub-fractions from *Withania aristata* leaves.

Extract/Fractions	*L. amazonensis* IC_50_^b^ (µg/mL)	*L. donovani* IC_50_ (µg/mL)	*T. cruzi* IC_50_ (µg/mL)	Murine Macrophages CC_50_^c^ (µg/mL)
Acetone extract	2.87	20.25	12.78	42.63
Fraction 1	12.27	19.12	28.91	81.39
Fraction 2	1.02	12.73	1.12	11.22
F2A	1.37	>50	11.24	13.79
F2B	1.05	3.06	1.93	8.02
F2C	1.40	4.21	3.14	10.23
F2D	0.36	1.85	1.73	7.65
F2E	0.19	2.70	2.02	13.90
F2F	2.11	>50	5.74	21.12
Fraction 3	4.53	22.28	3.63	46.11
F3A	2.51	9.03	8.42	22.90
F3B	2.48	5.96	6.04	23.78
F3C	2.35	>50	5.56	16.08
F3D	1.01	3.67	3.59	9.56
F3E	3.42	>50	7.05	21.57
F3F	19.52	>50	>50	43.98
Miltefosine ^d^	2.64	1.35		29.42
Benznidazole ^d^			1.81	104.1

^a^ Sub-fraction not included (F2G) in the table was inactive (IC_50_ > 50 µg/mL) against the three parasite species. ^b^ IC_50_: concentration able to inhibit 50% of the cells. ^c^ CC_50_: concentration able to reduce the cell viability by 50%. ^d^ Drugs used as the positive controls.

**Table 2:** Activity of withanolides **1**–**3** against the promastigote stage of *Leishmania*, epimastigote stage of *T. cruzi* and cytotoxicity against eucariotic cells.

Cp	*L. amazonensis* IC_50_ (µM ± SD) ^a^	SI	*L. donovani* IC_50_ (µM ± SD)	SI	*T. cruzi* IC_50_ (µM ± SD)	SI ^c^	Murine Macrophages CC_50_ (µM) ^b^
**1**	0.83 ± 0.07	14.36	13.22 ± 1.16	0.91	1.02 ± 0.10	11.69	11.92 ± 1.08
**2**	27.37 ± 1.03	3.12	>50		14.68 ± 0.43	5.82	85.47 ± 6.00
**3**	2.82 ± 0.07	4.35	20.88 ± 1.33	0.59	2.41 ± 0.11	5.09	12.26 ± 1.16
M ^d^	6.48 ± 0.10	11.14	3.31 ± 0.11	21.81			72.18 ± 1.25
B ^d^					6.95 ± 0.50	57.54	399.91 ± 1.04

^a^ IC_50_: concentration able to inhibit 50% of parasites, expressed as µM ± standard deviation (SD). ^b^ CC_50_ concentration able to inhibit 50% of murine macrophages, expressed as µM ± standard deviation (SD). ^c^ SI: selectivity index (CC_50_/IC_50_). ^d^ M: miltefosine, B: benznidazole were used as the positive controls. Cp: Compounds.

**Table 3:** Activity of withanolides **1**–**3** against amastigote of *L. amazonensis*.

Compound	*L. amazonensis* IC_50_ (µM ± SD)	SI ^a^
**1**	0.055 ± 0.009	216.73
**2**	0.663 ± 0.075	128.91
**3**	0.209 ± 0.028	58.66
Miltefosine	3.12 ± 0.12	23.13

^a^ SI: selectivity index (CC_50_/IC_50_).

## References

[1] WHO (2019). Neglected Tropical Diseases. http://www.who.int/neglected_diseases/diseases/en/.

[2] Rao S.P.S., Barrett M.P., Dranoff G., Faraday C.J., Gimpelewicz C.R., Hailu A., Jones C.L., Kelly J.M., Lazdins-Helds J.K., Mäser P. (2019). Drug discovery for kinetoplastid diseases: Future directions. ACS Infect. Dis..

[3] Bustos P.L., Milduberger N., Volta B.J., Perrone A.E., Laucella S.A., Bua J. (2019). *Trypanosoma cruzi* infection at the maternal-fetal interface: Implications of parasite load in the congenital transmission and challenges in the diagnosis of infected newborns. Front. Microbiol..

[4] Nagle A.S., Khare S., Kumar A.B., Supek F., Buchynskyy A., Mathison C.J.N., Chennamaneni N.K., Pendem N., Buckner F.S., Gelb M.H. (2014). Recent developments in drug discovery for leishmaniasis and human african trypanosomiasis. Chem. Rev..

[5] Cheuka P.M., Mayoka G., Mutai P., Chibale K. (2017). The role of natural products in drug discovery and development against neglected tropical diseases. Molecules.

[6] Eich E. (2008). Solanaceae and Convolvulaceae: Secondary Metabolites: Biosynthesis, Chemotaxonomy, Biological and Economic Significance.

[7] Chen L.X., He H., Qiu F. (2011). Natural withanolides: An overview. Nat. Prod. Rep..

[8] Cruz S.J. (2007). Más de 100 Plantas Medicinales. Medicina Popular Canaria Monografías.

[9] Llanos G.G., Araujo L.M., Jiménez I.A., Moujir L.M., Vázquez J.T., Bazzocchi I.L. (2010). Withanolides from *Withania aristata* and their cytotoxic activity. Steroids.

[10] Llanos G.G., Araujo L.M., Jiménez I.A., Moujir L.M., Bazzocchi I.L. (2012). Withaferin A-related steroids from *Withania aristata* exhibit potent antiproliferative activity by inducing apoptosis in human tumor cells. Eur. J. Med. Chem..

[11] Benjumea D., Martín-Herrera D., Abdala S., Gutiérrez-Luis J., Quiñones W., Cardona D., Torres F., Echeverri F. (2009). Withanolides from *Whitania aristata* and their diuretic activity. J. Ethnopharmacol..

[12] Chirumamilla C.S., Perez-Novo C., Van O.X., Berghe W.V. (2017). Molecular insights into cancer therapeutic effects of the dietary medicinal phytochemical withaferin A. Proc. Nutr. Soc..

[13] Jiménez-Ruiz A., Alzate J.F., MacLeod E.T., Kurt L.C.G., Fasel N., Hurd H. (2010). Apoptotic markers in protozoan parasites. Parasites Vectors.

[14] Basmaciyan L., Azas N., Casanova M. (2019). A potential acetyltransferase involved in Leishmania major metacaspase-dependent cell death. Parasites Vectors.

[15] López-Arencibia A., García-Velázquez D., Martín-Navarro C.M., Sifaoui I., Reyes-Batlle M., Lorenzo-Morales J., Piñero J. (2015). In Vitro Activities of Hexaazatrinaphthylenes against *Leishmania* spp.. Antimicrob. Agents Chemother..

[16] Vandana D.R., Tiwari R., Katyal A., Pandey K.C. (2019). Metacaspases: Potential Drug Target Against Protozoan Parasites. Front Pharmacol..

[17] Chandrasekaran S., Dayakar A., Veronica J., Sundar S., Maurya R. (2013). An *in vitro* study of apoptotic like death in *Leishmania donovani* promastigotes by withanolides. Parasitol. Int..

[18] Chandrasekaran S., Veronica J., Sundar S., Maurya R. (2017). Alcoholic Fractions F5 and F6 from *Withania somnifera* Leaves Show a Potent Antileishmanial and Immunomodulatory Activities to Control Experimental Visceral Leishmaniasis. Front. Med. (Lausanne).

[19] Kuroyanagi M., Murata M., Nakane T., Shirota O., Sekita S., Fuchino H., Shinwari Z.K. (2012). Leishmanicidal active withanolides from a Pakistani medicinal plant, *Withania coagulans*. Chem. Pharm. Bull. (Tokyo).

[20] Nasser A., Al-Sokari S., Mothana R., Hamed M., Wagih M., Cos P., Maes L. (2016). In vitro antiprotozoal activity of five plant extracts from Albaha region. World J. Pharm. Res..

[21] Lee I., Choi B.Y. (2016). Withaferin-A a natural anticancer agent with pleitropic mechanisms of action. Int. J. Mol. Sci..

[22] Llanos G.G., Araujo L.M., Jiménez I.A., Moujir L.M., Rodríguez J., Jiménez C., Bazzocchi I.L. (2017). Structure-based design, synthesis, and biological evaluation of withaferin A-analogues as potent apoptotic inducers. Eur. J. Med. Chem..

[23] Perestelo N.R., Llanos G.G., Reyes C.P., Amesty A., Sooda K., Afshinjavid S., Jiménez I.A., Javid F., Bazzocchi I.L. (2019). Expanding the Chemical Space of Withaferin A by Incorporating Silicon to Improve Its Clinical Potential on Human Ovarian Carcinoma Cells. J. Med. Chem..

[24] Sen N., Banerjee B., Das B.B., Ganguly A., Sen T., Pramanik S., Mukhopadhyay S., Majumder H.K. (2007). Apoptosis is induced in leishmanial cells by a novel protein kinase inhibitor withaferin A and is facilitated by apoptotic topoisomerase I-DNA complex. Cell Death Differ..

[25] Salmerón M.L., Quintana-Aguiar J., De La Rosa J.V., López-Blanco F., Castrillo A., Gallardo G., Tabraue C. (2018). Phenalenone-photodynamic therapy induces apoptosis on human tumor cells mediated by caspase-8 and p38-MAPK activation. Mol. Carcinog..

[26] Sifaoui I., López-Arencibia A., Martín-Navarro C.M., Chammem N., Reyes-Batlle M., Mejri M., Lorenzo-Morales J., Abderabba M., Piñero J.E. (2014). Activity of olive leaf extracts against the promastigote stage of *Leishmania* species and their correlation with the antioxidant activity. Exp. Parasitol..

[27] Jain S.K., Sahu R., Walker L.A., Tekwani B.L. (2012). A parasite rescue and transformation assay for antileishmanial screening against intracellular *Leishmania donovani* amastigotes in THP1 human acute monocytic leukemia cell line. J. Vis. Exp..

[28] Fadel H., Sifaoui I., López-Arencibia A., Reyes-Batlle M., Hajaji S., Chiboub O., Jiménez I.A., Bazzocchi I.L., Lorenzo-Morales J., Benayache S. (2018). Assessment of the antiprotozoal activity of *Pulicaria inuloides* extracts, an Algerian medicinal plant: Leishmanicidal bioguided fractionation. Parasitol. Res..

[29] Codonho B.S., Costa S.S., Peloso E.F., Joazeiro P.P., Gadelha F.R., Giorgio S. (2016). HSP70 of *Leishmania amazonensis* alters resistance to different stresses and mitochondrial bioenergetics. Memórias do Instituto Oswaldo Cruz.

